# The American and Colonial Journals: Medical Statistics

**Published:** 1839-10

**Authors:** 


### MEDICAL STATISTICS.

*Classification of Fracture Cases treated at the Native Hospital, Calcutta, from* 1815 *to* 1837. *Communicated by* J. R. Martin, Esq., *Surgeon to the Institution.*

| | |
|---|---|
| Fractures of the skull | 70 |
| Ditto nose | 12 |
| Ditto lower jaw | 26 |
| Ditto cervical vertebræ | 2 |
| Ditto clavicle | 59 |
| Ditto scapula | 2 |
| Ditto acromion process of the scapula | 1 |
| Ditto sternum | 8 |
| Ditto ribs | 64 |
| Ditto spine, with displacement | 9 |
| Ditto of the pelvis | 2 |
| Ditto humerus | 235 |
| Ditto of the condyles of the humerus | 2 |
| Ditto radius and ulna | 340 |
| Ditto into the elbow-joint | 9 |
| Ditto into the wrist | 20 |
| Ditto of the metacarpal bones | 2 |
| Ditto fingers | 10 |
| Ditto neck of the thigh-bone | 14 |
| Ditto trochanter major | 1 |
| Ditto thigh-bone | 292 |
| Ditto patella | 22 |
| Ditto tibia and fibula | 343 |
| Ditto into the ankle-joint | 9 |
| Ditto of the metatarsal bones | 6 |
| Ditto of the toes | 12 |
| Total | 1572 |

*Diseases of House-Patients treated at the Native Hospital, Calcutta, from Sept.* 1, 1800-1, *to Aug.* 31, 1836-7.

| | |
|---|---|
| Diseases of the digestive organs | 273 |
| Ditto respiratory ditto | 97 |
| Ditto circulation | 2 |
| Ditto brain and nervous system | 344 |
| Ditto generative and urinary organs | 504 |
| Febrile diseases | 1547 |
| Cholera | 540 |
| Dysentery | 310 |
| Rheumatism | 533 |
| Dropsical diseases | 303 |
| Cutaneous ditto | 43 |
| Various fractures and other injuries requiring operations | 7112 |
| Hydrocele cured by radical operation | 162 |
| Ulcers | 1382 |
| Miscellaneous cases, chiefly surgical | 1372 |
| Total | 14,524 |

N.B. It is proper to mention, for the information of strangers to the nature of the institution, that it has but 50 beds, and was founded solely for the relief of "persons suffering from accidents," and that medical cases are admitted only when the condition of the wards admits of it. A dispensary is attached to the hospital, affording relief to an average of seventy-five thousand per annum.

*Calcutta Quarterly Journal. January,* 1838.

